# American Dental Association

**Published:** 1875-01

**Authors:** 


### SELECTED ARTICLES.

### ARTICLE III.

*American Dental Association.*—CONTINUED.

THIRD DAY.—*Morning Session.*

Called to order at ten o'clock, President Buckingham in the chair.

Dr. Rehwinkel, chairman of the committee appointed last year to endeavor to procure the appointment of dental surgeons in the army and navy, reported that they had concluded that such proposed action was premature; the tendency of Congress is to cut down expenditures, and it was deemed expedient to let the matter rest for a time. The report was accepted, and the committee discharged.

The subject of "Operative Dentistry" being still open, Dr. Cushing said that at the meeting of this Association three years since, a paper was read on "Light vs. Heavy Foil," in which the author, Dr. Abbot, claimed to establish by experiment the fact that a given cavity would contain more foil of a light number than of heavy. He had felt confident that this conclusion could not be true, and that the experiments had not been fairly made; nor could they be so when performed by the same person, who was not an expert in the use of one method, while he was in another. He had therefore instituted some experiments with the same instrument. He had requested Dr. Allport, whose capabilities in packing soft foil would not be questioned, to fill the cavity in the steel plate twice; once with soft foil unannealed, and once with adhesive foil annealed. Dr. Allport had done so, packing the first with Abbey's No. 4 unannealed, with hand pressure, which he designated as an "old fogy" filling; and the second with the same foil, annealed, packed with the mallet, in the usual way. The soft and adhesive foil fillings weighed exactly the same, seventeen and a half grains each.

The speaker had also made some fillings in the same cavity, with results as follows: No. 1, with Ashmead's No. 3 cohesive foil, small pellets, annealed as used, with fine pointed plugger, weight fifteen and a half grains. No. 2, Williams' rolled gold, No. 120, annealed as before, larger pointed instrument, weight nineteen and a half grains. The speaker submitted these experiments that a record might not go out from this Association that would lead to erroneous conclusions. For himself he preferred the heavier foils, and believes that in skillful hands they produce better results, on the whole, than lighter or soft; yet agrees with Dr. Allport that the quantity of gold in a cavity is not of so much importance as that it should be properly packed against the walls.

Dr. Butler thinks there is more time spent now on some classes of gold fillings than was demanded twenty years ago; an entirely different class of operations is now called for. If we spend more time, it is a necessity; many large fillings are now made that were not thought of a few years ago. People like fine things; there is a greater degree of refinement, and it runs through all their wants; they must go into luxuries as well as necessities, and fine fillings, even as far back as the third molar, are absolutely demanded. In the arts, fine productions must take time; a fine piece of work can be made into form quicker than it can be finished. It is the same with fillings.

As to rubber dam, why is thin the best? Thick is sometimes necessary, and causes no more pain. It is a mistake about wasting time putting it on; it is time saved. There is a certain class of patients for whom he could not be induced to operate, if he could not use rubber. In regard to root filling, it is hard to conceive how a substance that is not plastic can be introduced as perfectly as a plastic material. If osteoplastic is carried in with the gold, it can be forced into every inequality more perfectly than gold. There is no rule for the kind of gold. Every one for himself on that question ; but when restoration is attempted, cohesive must be used ; but it is not certain that extra-cohesive will give good results. The walls are apt to be powdered. If soft foil were used, it could be made perfectly tight. He uses no higher number than 66 ; if confined to one preparation, would use Williams' No. 30.

Prof. Knapp. Is pleased with the report. It is easy to criticise; let those who do, try to make a better. Does not agree, however, as to the credit to which Dr. Arthur is entitled in the introduction of adhesive foil. Old members of the profession will remember that thirty years ago Leach made gold more adhesive than we have at present. There is far too much adhesive gold used to-day. He does not disparage but deprecates its indiscriminate use. It requires greater pressure, and soft is more safely used in frail teeth. It is not the amount of gold that is essential to preserve the teeth. It pleases the younger men to assert that fine work cannot be done without the dam, mallet, and adhesive foil ; they are all good things in their place, but cavities on the labial surface have been excellently filled without the dam. Even cavities under the gum, unless extreme and bleeding, may be thoroughly filled and kept dry. When he sees skilled operators fail, he is led to inquire why we should put the patient to the expense of money and time necessary. If the cavity is properly prepared, and the gum treated two or three days, napkins would be sufficient. Rubber is not pleasant, and is carried to an unwarrantable extreme. Machinery is too frequently resorted to, especially by unskilled operators ; too much is cut away ; when the pulp is exposed there is great danger. Do we fill teeth to make jewelry, or to preserve them ? They were preserved before adhesive gold or the mallet were used. The time, pain, and expense necessary do not warrant us in using these things indiscriminately. Had known bad effects such as inflammation of the periosteum, from wedging and mallets.

Dr. John Allen said the report dealt fairly with the subject. We have great facilities at the present day, and the wonder is that as good work was made in old times as was made. As to the different methods, if our operations are good, it makes but little difference how we do them. Some will fail with soft foil, others with adhesive, and others will fail anyhow. The balance of testimony is in favor of soft gold. He can look back forty years and call to mind many fillings that are yet doing service. There are many outside of the profession who have aided us materially, and we should give them credit for the various manufactures and improvements in teeth, gold, and materials. Dental science is advancing, with the exception of the artificial branch; in that we have retrograded.

Dr. Keely thinks that, with heavy foil and the mallet, we can pack to frail walls with greater certainty, ten to one, than by hand pressure and soft foil, and can lap the gold over and protect the enamel. With the engine, heavy gold, mallet and dam, better operations are made. If every one asks blessings upon the head of the inventor of the dam as often as he does, he will be abundantly blessed.

Dr. Kulp said that he occupied a peculiar geographical position, and saw operations from all parts of the country. His opinion, based upon what he has seen, was that those who use the dam and the heroic practice of dressing out cavities, and heavy foil, are the ones whose fillings show the best. There is very little danger of cutting away too much. We vacillate too much, to-day using one thing, and to-morrow another. We should stick to one thing and have uniformity. Had tried all numbers, from 2 to 120, and uses numbers 4 and 60. In building up, uses large instrument and No. 4 foil, which takes much less time. The dam is less objectionable than the wedge.

Dr. Rehwinkel. All our arguments now seem to tend against the abuse of good things. The report gives a faithful *resume* of the subject. Different modes must be left to individuals. Operations can be and have been done without the dam, but those who did them would not now do without it. They find themselves relieved of mental anxiety in filling. It has been said, in fact, that a great objection to rubber is the habit it produces of leaving the patient half an hour to converse with others in the next room. There are many in the profession who could not fill a tooth without rubber or adhesive foil. The preceptor does not do his duty who allows a student to go out without instruction in both. Any young man who will learn to fill well with soft foil, will soon learn to do so with cohesive. As to root filling, if an outsider were to come in here, what would he think? All the advocates of different modes claim ninety-nine cases out of a hundred. No one tells you that the condition of the tooth or system has anything to do with it. It is the *sine qua non*. Now (to Dr. Wetherbee,) you said that gold was the best under all circumstances; when you find it impossible to remove all the pulp, is gold the best for that case?

Dr. Wetherbee. I remove it entirely in incisors, cuspids, bicuspids, and molars, in from ten to twelve hours in all cases.

Dr. Rehwinkel. In every case?

Dr. Wetherbee. Yes, sir; in every case.

Dr. Rehwinkel. You have very funny people in Boston. (Laughter.)

Dr. Wetherbee. We have very sensible people there, and, beside, they have a dentist who knows his business! (Laughter and applause.)

Dr. Rehwinkel. He ought to add that the pulps of their teeth have sense enough to get out when they have no business there! People west of the Alleghanies are not so sensible, and their pulps stay as long as they can, and have to be removed by *hook and crook!* (Laughter and applause.)

Dr. Stockton. Teeth can be filled with each kind of foil that cannot be with the other. Uses an equal amount of both soft and adhesive, finishing with heavy. Some teeth he can fill more quickly with soft foil. We must not have our hobbies. Instruments and appliances are so perfect that operations should not be defective. He had repeated the ink experiments of Fletcher, of England, with entirely different results, Dr. Osmond asserts that after a filling has been malleted tight, it may be malleted loose, and Fletcher probably committed that mistake. The condition of the tooth has something to do with the choice of material. If the walls are frail, would not use gold, as the walls will be broken up. You cannot build a good house on a poor foundation.

Dr. Bogue. Fletcher has recalled his statements, and admits that tight fillings can be made with adhesive foil. Dr. Kulp's experience is valuable. We do not realize the difference that exist in teeth and in individuals. Instead of becoming a degenerated race, we are absolutely improving. Such children as would formerly have died are now reared. The same is true with regard to teeth; they were formerly indiscriminately slaughtered. The old fillings we hear of are generally simple crown cavities. Such fillings as are now inserted were not in existence. Filling cavities is different from building up crowns. Soft foil and napkins and a little labor will do one, but not the other. He was glad to see a return to the easy ways, where they were effective.

Dr. Judd. The spirit of the report has not been touched upon. The idea was that our practice should be so modified as to afford operations at a less price, that numbers now unable could avail themselves of them. There are conditions about this in large cities; there is there a class of people able and willing to pay for the best operations, and they will be satisfied with nothing less; they will patronize those who do work according to their notions. There are other operators who spend less time and labor and skill, and they will be surrounded by another class of patients; and so down to the man who slips into the five-dollar establishment for a set of teeth. Every grade is demanded. The country practitioner must fill all these requirements. The quick operator gets careless. The report was too conservative about the dam. It recommended the mean between two extremes, but in this case leaned from the median line. In long operations we should never dispense with it. Patients are glad of the rest it gives; confidence is given to the operator. Fletcher's experiments are not worthy of attention. The recommendation of tin, in large cavities, does not meet with his (the speaker's) full indorsement. It saves simply a small amount of gold. He does not sell gold for a living, but charges for his time and skill and experience. Soft gold works as easily as tin.

In some cases too many fissures are cut out, but many operators do not cut out enough. Fissures are natural in many cases. Recommends cutting out so far as there is any indication of decay, but it is time enough when decay shows itself. Believes in filling canals with gold; there is no better material. Is gratified that there has been no cotton advocated in this discussion; it is a mark of progress. As to the possibility of filling all roots to the apex with gold, it can't be done. Has examined many specimens, and in many instances no instrument can be carried to the apex. (Dr. Wetherbee here stated that he did not intend to claim that all canals could be filled to the apex with gold.) As to opening them up by drills, it was a perfect failure, as a general thing; the drill was apt to run out at the side, He had seen a cuspid so drilled by a dean of a dental college (so called.) He (the speaker) had filled it with oxychloride some years ago, and it was doing well. Is glad to hear a refutation of Dr. Abbott's experiments. We can fill teeth with adhesive foil and mallet, that are too frail to stand hand pressure. Many fine operators are very poor dentists. We have more disease from injudicious operations than from all other causes. Pulps seem to die more readily under amalgam than gold.

Prof. Shepard. The report has been misunderstood, but it has furnished a stimulus for discussion. The principle of it is this,—a great many people are poor, and are sufferers; the great majority of dentists must be dentists to these poor ones. To those who minister to the rich, the expense of material is nothing, but the dentist to the poor must get pay for $3 or $5 worth of gold. The dentist who gets $1 per cavity is just as respectable and just as much a benefactor as the one who gets $10. This point is too much ignored. Prominent men have been considered to be those who get high fees, and not those who do most good. This last should be the standard. Country dentists are in the majority. The main objections to the report have been in regard to omissions; but telling how this or that is done not properly come into this body. We would give credit to whom it is due; if Dr. Arthur had cared to take out a patent for adhesive gold in 1855, he could have done so, but he had published the thing abroad, and should have credit for it. It is almost impossible in college instruction to get students to care anything about learning to use soft foil or to fill a tooth without the dam. This is a great error.

Dr. McQuillen. The fees of a dentist do not give him reputation and position; it is the character of his operations. There is danger of cheap operations for the poor misapprehended. Some of the best operators in the cities are supplied from the country. The talent of the village seeks the city, to enjoy the fruits of a reputation made by performing good operations for a small compensation. The best work should be done under all circumstances. He was opposed to the practice of filling roots with cotton. With respect to fissures in the enamel, the finest probes should be used in making an examination of the teeth, and where such an instrument catches, the place should be cut into and filled. These fissures, microscopical in size, often lead to dentine in which the interglobular spaces are abundant, or otherwise defective in structure, and sometimes they open into cavities in which caries has accomplished its work of destruction without presenting any external evidence of the fact.

Dr. Judd explained that what he said was that there were fissures upon every molar tooth; they are natural, and a part of its anatomy, and unless there are indications of delay, it is not best to cut them out under all circumstances.

Dr. Allport. Had noticed the experiments of Fletcher. No doubt some operators can pack cohesive gold sufficiently well to preserve the teeth; but it is not what a few of the best operators can do; it is the kind of gold best adapted to the skill of the mass of operators that should be generally adopted. It is not necessary to have Fletcher's experiments to prove that cohesive gold fillings leak. One-half of all the cohesive foil fillings are discolored; they are beautiful but worthless. The old-fashioned fillings of Harris, Maynard, and others, though so soft that you can stick a plugger through them, are to-day absolutely preserving the teeth; they are scooped out, but are saving the teeth. Why? Because the material was better adapted to the average skill. In some cases cohesive gold is necessary; there is not the same danger of fracturing the walls of a frail tooth, because it is not driven directly against them; when it is used, however, you should pack soft gold against the walls. Cohesive gold is very deceptive; you think you have a beautiful filling, but it will fail with ordinary operators. Did not use the rubber for a good while, but now finds the places where it is most difficult to apply, the very places where it is wanted, and can hardly do without it. As to fissures, there are natural fissures with no presence of decay; you may catch your instrument in them, and at the same time find no decay. When the tooth is decayed, it is time enough to fill it; but if the teeth are soft, it is safe to anticipate it.

Dr. Bogue, chairman of the committee appointed for the purpose, reported the following memorial resolution, which was unanimously adopted:

*Whereas*, This Association has with sorrow learned of the death of Prof. Thomas B. Hitchcock, of the Harvard Dental School, one of our most valuable members, and the chairman of the Committee on Histology and Microscopy; therefore,

*Resolved*, That we signify in this public manner our sense of the great loss which the profession and the cause of dental education have sustained, and that this resolution be inserted in the records, in the place where the report of the committee would have appeared.

Adjourned.

*Afternoon Session.*

The subject of "Operative Dentistry" still under discussion.

Dr. S. B. Palmer said that tin foil had antiseptic properties. There is a chemical or galvanic action between all the metals we put in the mouth, and even between gold and tooth-bone. Chemical and galvanic action are synonymous. It has been generally supposed that bone, not being a conductor, no action existed between it and the metals; but when the galvanometer is applied, it is found to be otherwise. He cracked a tooth and put it with gold into a dilute acid, and found that the tooth was being consumed. In an alkaline condition, the action is reversed. If the gold is pure, so much the worse for the tooth. The tin filling being in a loose porous condition, is itself consumed, while the tooth is preserved. For this reason, prefers it in the sixth-year molars, or in children's mouths, because it renders the tooth negative till it will stand the wear.

Dr. Walker. For operators outside of Boston, to remove all the nerve in every case is impossible. In root-filling it is not necessary to resist mechanical or chemical action but simply to use something that will occupy the space and has an antiseptic property. The fissures in the canals can be better filled with Guillois's cement than with gold. Uses it a little soft, and pushed up with cotton.

Prof. Knapp. In cases of bleeding after extraction, which are not controlled by the ordinary means, as in hemorrhagic diathesis, cut a plug of wood resembling the roots cutting a few creases in it, around which wind a little cotton saturated with the perchloride of iron. In this way the most obstinate hemorrhage is easily checked.

Dr. Atkinson. The bleeding, in such a case, is not from a hemorrhagic diathesis, but from a broken artery which is attached so that it cannot contract. All you have to do is simply to take a bur and give it one turn in the bottom of the socket, and kiss your patient good-by! Criticism is a two-edged sword; it is not fault-finding, but it is a test of truth. Admires the spirit of the report, and would only suggest that we give in such reports less of individual opinion, and more of principles. The mechanism of filling teeth has entered into this discussion more than the principles involved. At the cervical walls an immense preponderance of failures occurs. That is where we need the greatest care. What constitutes this? Keeping the cavity so that we can see. How anybody can fill under the gum satisfactorily without the dam, is at a loss to see. Cut away the excess of gum, instead of wedging back with cotton; drill a hole above the cavity and put in a screw, and hook the dam over it, and file and finish off the screw when you do the filing. Use whatever means pleases you, but, by all means do good work. He has not backslidden any farther than to go back to No. 30, since the introduction of heavy foil. It is a saving of time sometimes to be slow. Put on the rubber without getting excited. Only a small minority obey the law of contour; until that is done, there is no perfect dentistry. The teeth are analogue in their position to the staves of a barrel, and you cannot afford to lose one of them. Even the wisdom-teeth are proper abutments, and the breaking of the arch is where the mischief comes in. There is no difference of opinion about good cheap dentistry. Cheap is not low-priced; shoddy is not cheap. He has yet to see the first man that does not ask enough for what he does.

Dr. Webb. Instruments of almost every conceivable form, with the smooth, plain, or convex point, along through various grades, of serrations, the broken point, and then the alloyed gold points,—all have been used and eulogized. The cohesive property of gold was recognized, and then too Dr. W. H. Atkinson introduced and advocated the mallet as a valuable aid in the impactation of the precious metal, and both were adopted.

To-day the cohesiveness of absolutely pure gold is deemed quite sufficient for all purposes as a filling, except that it is best to pass it through the flame of alcohol when the impactation is to be carried to a restoration of contour.

To-day, also, we have electricity applied as a force for the impactation of gold, and those who have used that representation of true genius—the improved electro-magnetic mallet—cannot but feel thankful to the inventor for it.

The lightning like blows impact gold to a greater degree of perfect solidity than has otherwise ever been accomplished.

Dr. Palmer asked how labial cavities were to be kept dry without the dam.

Dr. Shepard. Get everything ready on the table. Use a rope of bibulous paper under the lip. Don't allow closure of the mouth; use care not to wound the gum. Had filled such cavities which had failed under the best operators, and the patients stated that they had experienced less pain.

Prof. Taft. The preservation of the pulp is a very important matter, and one of the most difficult things a dentist is called upon to accomplish. He is sad and sorry to hear of destroying pulps—poisoning them to death. We must get over this impression. In many persons the destruction of the pulp is equivalent to the loss of the tooth, and under most favorable circumstances the loss of the pulp insures the loss or detriment of the tooth. Its course is onward to the destruction of the tooth, and this ought to be engraved with a pen of iron upon the mind of every dentist. A great many say they cannot save the pulp, but a great many do save them,—in almost all cases. Some fail, but what physician poisons his patients because he cannot save them all? We should recognize the importance of the organ, and save every pulp possible, and make an effort to save every one. How shall it be done? Pursue a rational treatment as with other tissues. Knowledge will enable us to know what to do. If the pulp is in a condition of comparative health, it should then be the aim simply to close it up. Cover it with some substance that will be acceptable to it, that will not decompose, will not produce pressure, and is a non-conductor,—seal it up, and all will be well, If it has been exposed or diseased, the treatment will depend upon the condition of the system. Dr. Rehwinkel says, in his neighborhood they have so much malaria that they have to take quinine by the ringing of the bell, and he can't save pulps in that locality. Systemic conditions must be recognized.

Dr. Thomas agrees with Prof. Taft as to the propriety of efforts to save every pulp. But what about teeth that we find remaining in the mouth after the pulp is destroyed,—dead teeth as they are called? They have given him a great deal of trouble. Within the last two years he had excavated and cleaned thoroughly, and filled at once, and then made an opening through the gum with a lance to the apex of the root, cutting down the bone with a trepanning instrument.

Dr. Rehwinkel is as anxious as any one to save the pulp; but there are cases where we can serve patients better by destroying it in the most harmless manner. It is not in his opinion wrong, but good practice, to resort to arsenic, and he uses it because he don't want to be two weeks about it, and has yet to see the first case of bad effects clearly traceable to its use.

Dr. Taft. The same treatment will not serve in all cases. Local treatment will be required, and the first step is to clear away the debris, remove all irritating matter, and correct the offensive condition by a disinfectant. It is difficult to give a formula for all cases; carbolic acid, chloroform, permanganate of potassa, etc., may be employed. It is not always good to deplete—sometimes it is well, but sometimes bad. Then use stimulants, tonics, astringents; the object being to relieve the part, and bring it to a state of health. Pulps may sometimes be saved where there is suppuration. Uses simply oxide of zinc with creasote, and over that oxychloride; afterwards fills with gold. Within the last two or three months, had used lacto-phosphate of lime, or lactic acid and phosphate of lime, which can be kept without deterioration. Mixes them into a paste and applies to the pulps in the same way as the oxide of zinc and creasote.

Dr. Allport. Though he has not tried this preparation, has tried treating a pulp in his own person with satisfaction. The treatment of the pulp is the same as that of other parts of the body. For chronic periostitis has used, in a large majority of cases, simply aconite of high potency,—the third dilution (homœopathic,) as many pellets as can be put on a three-cent piece. Do not give too much, and stop as soon as the patient begins to get better. Usually two doses will suffice. Has been astonished at his success with it. Formerly had used mercurius. In troublesome cases, alternates with antimony.

Dr. Hunter had accomplished the same result with a few drops of tincture of aconite, applied in water, locally.

The subject was then passed.

The Committee on Appliances made a report, of which the following is a synopsis:

The committee merely mentioned the articles brought to their notice, among which were—

A vulcanizer, with a self-packer, and a gravity safety-valve. A floss-silk holder for the pocket or office use. An instrument for separating teeth, devised by Dr. O. A. Jarvis, a valuable adjunct in operative dentistry; a tongue and cheek protector, used in connection with the corundum disk on the engine; separating files with corrugated handles, rendering them more easily held between the fingers; a triplex dam-punch, simple and convenient. Improved retaining screws with split heads, which give them an advantage over the ordinary screw. A set of four scalers, worthy of attention. Some forceps, burs for engine, and excavators, apparently of fine material and excellent temper. A patent process for packing rubber, which was considered simple and effective. The S. S White dental engine, containing valuable improvements as regards the pitman and flexible arm, running smoothly and noiselessly, and allowing a perfect control of the hand-piece in operating; an hydraulic lift attached to the Harris chair, which accomplishes the desired end with very little effort; an air cushion in the back of chair; also a changeable seat and extension back; a vulcanizer so designed that it may be set at any given pressure, and, so set, will not vary from this point; also, Green's electric engine as improved by S. S. White, heartily recommended to those not employing an assistant; some hard rubber disks, to be used in polishing approximal fillings, highly spoken of by those who have used them.

An electric plugger was exhibited in operation: a beautiful instrument, with no apparent deficiency of power.

An improved vulcanizer, containing an arrangement for closing the flasks after being placed in the vulcanizer, and that without producing any strain on the bottom.

The subject of "Etiology" was then again taken up, and Dr. McQuillen made a report. (This report will be published in a subsequent number of the Dental Cosmos.)

Dr. Noel called attention to the action of lactic and acetic acids in the production of decay. Saliva, has, as is well known, the power of converting phosphate of lime into grape sugar. Cane sugar is often present in the mouth and this, by appropriating the elements of water, may be converted into grape sugar. This is composed of the elements carbon, hydrogen, and oxygen; and by a heat of 98° may be decomposed into lactic acid, which immediately attacks the dentine. Acetic acid may also be generated. The tribasic phosphate of lime, of which the dentine is composed, is converted into the bibasic, and can then be washed away.

Adjourned.

*Evening Session.*

The Association was called to order at the usual time by the president, the special business being the selection of place of next meeting, and election of officers.

Upon the first ballot for next place of meeting, Niagara Falls, the only place nominated by the committee, was selected.

The election of officers was then proceeded with, with the following result:

*President.*—M. S. Dean, Chicago.

*First Vice-President.*—G. W. Keely, Oxford, Ohio.

*Second Vice-President.*—Jas. S. Knapp, New Orleans, Louisiana.

*Corresponding Secretary.*—G. L. Field, Detroit.

*Recording Secretary.*—C. Stoddard Smith, Springfield, Illinois.

*Treasurer.*—W. H. Goddard, Louisville, Ky.

*Executive Committee.*—(New members) A. H. Brockway, G. C. Daboll, and S. B. Palmer.

Adjourned.

TO BE CONTINUED.

