# Correction

**Published:** 1875-02

**Authors:** 


*Correction.*—EDITOR JOURNAL.—*Dear Sir*: In your report of the proceedings of the American Dental Association, (Jan. No. page 409,) your reporter makes me say, "Saliva has, as is well known, the power of converting phosphate of lime into grape sugar."

I said Saliva had the power of converting STARCH into grape sugar.

You will greatly oblige me by making this correction in the next issue of the Journal.

Respectfully and truly yours,

*Nashville, Tenn., Jan.* 13*th*, 1875. L. G. NOEL.

---

### MONTHLY SUMMARY.

